# MORC protein family-related signature within human disease and cancer

**DOI:** 10.1038/s41419-021-04393-1

**Published:** 2021-11-27

**Authors:** Huan Wang, Ling Zhang, Qiuhua Luo, Jia Liu, Guiling Wang

**Affiliations:** 1 Department of Cell Biology, Key Laboratory of Cell Biology, Ministry of Public Health and Key Laboratory of Medical Cell Biology, Ministry of Education, China Medical University, Shenyang, 110122 China; 2 Department of Biotherapy, Cancer Research Institute, The First Affiliated Hospital of China Medical University, Shenyang, 110001 China; 3 Department of Pharmacy, The First Affiliated Hospital of China Medical University, Shenyang, 110001 China

**Keywords:** Cell death, Targeted therapies

## Abstract

The microrchidia (MORC) family of proteins is a highly conserved nuclear protein superfamily, whose members contain common domain structures (GHKL-ATPase, CW-type zinc finger and coiled-coil domain) yet exhibit diverse biological functions. Despite the advancing research in previous decades, much of which focuses on their role as epigenetic regulators and in chromatin remodeling, relatively little is known about the role of MORCs in tumorigenesis and pathogenesis. MORCs were first identified as epigenetic regulators and chromatin remodelers in germ cell development. Currently, MORCs are regarded as disease genes that are involved in various human disorders and oncogenes in cancer progression and are expected to be the important biomarkers for diagnosis and treatment. A new paradigm of expanded MORC family function has raised questions regarding the regulation of MORCs and their biological role at the subcellular level. Here, we systematically review the progress of researching MORC members with respect to their domain architectures, diverse biological functions, and distribution characteristics and discuss the emerging roles of the aberrant expression or mutation of MORC family members in human disorders and cancer development. Furthermore, the illustration of related mechanisms of the MORC family has made MORCs promising targets for developing diagnostic tools and therapeutic treatments for human diseases, including cancers.

## Facts

- MORC family members share common domain architectures, but each protein exhibits unique structural motifs, which are responsible for their diverse biological functions.
- MORC family members are shown to have specific cell and tissue distribution. They mainly localize to the nucleus, with a small percentage located in the cytoplasm and other subcellular compartments in response to epigenetic signals.
- The aberrant overexpression of MORC proteins positively correlates with high degree of malignancy and short overall survival of cancer patients, therefore, MORCs are regarded as promising therapeutic targets for the diagnosis and treatment of tumors.
- Clinical and prognostic signatures comprising MORC proteins vary among different cancers.
- Mutations in MORC proteins were observed in not only cancers but also other diseases, such as neurogenic disorders and metabolic bone disease.

## Open questions

- The underlying mechanism by which MORC proteins modulate disease and cancer remains largely unknown.
- The precise role of aberrant expression and mutation of certain MORC proteins in specific diseases and cancers deserves to be studied.
- The role and mechanism of the subcellular localization of MORC proteins have not been well defined.
- Considering the promising therapeutic roles of MORC proteins in disease and cancer treatment, the potential side effects related to the modulation of the MORC protein family need to be carefully investigated.
- The novel role of MORCs in regulating noncoding RNA remains largely unknown.

## Introduction

Over the past decade, advances in genomics have led to a boom in the discovery of new genes, among which are the microrchidia (MORC) genes. Although MORC proteins have garnered relatively little attention, they have recently entered the spotlight owing to their aberrant expression and mutations in cancer and some diseases. MORCs compose a highly conserved nuclear protein superfamily and have been identified in diverse eukaryotes, including plants, animals, and humans [1]. MORC, which was first characterized as a regulator of testis formation and germ cell development in males in 1996, is now named as MORC1 [1, 2]. Decades of research that followed revealed other MORC members and several of their key structural and biological aspects of biology, such as common domain structures (GHKL-ATPase, CW-type zinc finger, and coiled-coil domain) and diverse biological functions. In addition, MORC proteins were shown to have their own specific pattern of cell and tissue distribution.

To date, four MORC family members have been identified in mammals: MORC1 (MORC, ZCW6 or CT33), MORC2 (ZCW3, ZCWCC1, KIAA0852 or AC004542), MORC3 (ZCW5, ZCWCC3, NXP2 or KIAA0136) and MORC4 (ZCW4, ZCWCC2, FLJ11565 or Dj75H8.2) [3]. Human MORC proteins can be further classified into two subfamilies according to the structure of CW domain; MORC1 and MORC2 belong to subfamily I, while MORC3 and MORC4 are classified as subfamily IX [4, 5].

The research of the MORC family initially emphasized their role in regulating testis formation and germ cell development [1, 2], with later studies focusing on activities relating to epigenetic regulation and chromatin remodeling [3–5]. To date, MORC members are regarded as oncogenes, whose overexpression is related to the high degree of malignancy and short overall survival in several cancer types, including breast cancer, liver cancer, and others [6–8]. In addition, a variety of mutations in MORC proteins were observed in tumors and other diseases, such as neurogenic disorders and metabolic bone diseases. Taken together, these data suggest that the aberrant expression and mutations increase the activity of MORC proteins, resulting in genomic instability and dysregulation of target genes, which is thought to ultimately lead to human disease and tumors [2, 9, 10]. This new complexity of MORC function could shed light on cancer diagnosis and treatment.

In this review, we compile a comprehensive list of the domain architectures and distribution characteristics and draw from recent literature to discuss the emerging roles of MORC proteins in human disorders and cancer development. Furthermore, we studied and predicted MORC function and its future value as a potential target for the diagnosis and treatment of multiple diseases.

## Domain structures and functions

**Fig. 1:**
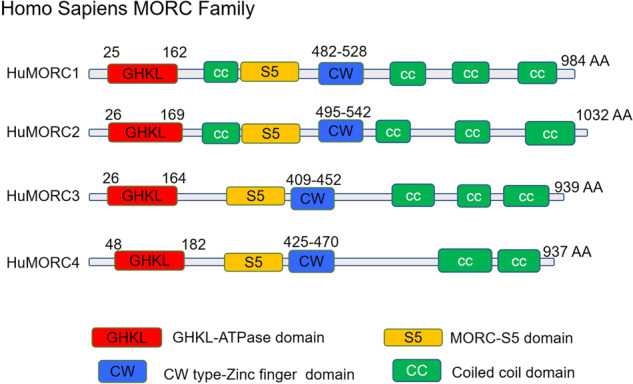
The arrangement of the domains of human MORC family. According to given evidence and Pfam analysis results, the domain structures in human MORC protein have been defined with three hallmarks: a conserved GHKL-ATPase domain at N-terminal, a conserved CW-type zinc finger domain in the middle and several coiled-coil domains. In addition, MORC proteins in eukaryotes contain a structure similar to that of the second domain of ribosomal S5 proteins (hence named the MORC-S5 domain). Aside from these common structural determinants, human MORCs also exhibit unique structural motifs, such as nuclear matrix binding and RNA bindings, which are particularly apparent in MORC3.

MORC is short for “MORC family CW-Type Zinc Finger: or “Zinc Finger CW-Type Coiled-Coil Domain Protein”. Evidence and analysis results from the Pfam study indicate that there are three hallmark domain structures in MORC proteins: a conserved GHKL-ATPase domain at N-terminus, a conserved CW-type zinc finger domain in the middle and several coiled-coil domains [3] (Fig. 1). In addition, MORC proteins in eukaryotes contain a structure similar to that of the second domain of ribosomal S5 proteins (hence named the MORC-S5 domain), which is reported to regulate ATPase activity by forming a module complex with the GHKL-ATPase domain [11]. These domains are responsible for the roles of MORC in transcription regulation, chromatin condensation and remodeling, and DNA break repair (Table 1). Aside from these common structural determinants, human MORCs also exhibit unique structural motifs, such as nuclear matrix binding and RNA bindings, which are particularly apparent in MORC3 [12].

**Table 1:** Domain architectures and biological functions of MORC family.

Domain	Functions	Ref.
GHKL-ATPase domain	DNA damage response	[13]
	Genome compacting	[14]
	Gene regulation	[15]
CW-type zinc finger domain	Methylation recognition e.g. H3K4me3	[16]
	Nucleic acid binding	[17]
	Protein–protein interaction	[4]
Coiled-coil domain	Regulation of nucleic acid binding	[18]
	Regulation of protein–protein interaction	[19]
	Molecular recognition	[20]

### GHKL-ATPase domain

MORC proteins contain a GHKL-ATPase (gyrase or topoisomerase; G), heat shock (HSP90; H), signal transduction (histidine kinase; K) and DNA mismatch repair (MutL; L) domain that is required for catalytic ATPase activity, which is responsible for hydrolyzing ATP, regulating DNA binding and promoting chromatin remodeling in response to DNA damage [13, 14, 21, 22]. Additionally, it is indicated that the GHKL-ATPase domain can cooperatively interact with CW domain, which is involved in transcriptional modification and DNA binding. For example, the crystal structure of the ATPase and CW domains of MORC3 in complex with a nonhydrolysable ATP analog indicated that these two domains were directly coupled [23–25]. Similarly, the ATPase/CW domain interaction stabilizes the protein fold but inhibits the ATPase activity of MORC3. This interaction is disrupted by the binding of the CW domain to histone H3 which results in activation of the ATPase activity [24, 25]. In addition, the crystal structure of MORC3 showed that the ATPase-CW domain can bind to the nucleotide analog phosphoaminophosphonic acid adenylate ester (AMPPNP) and form a complex with a trimethylated histone H3 lysine 4 (H3K4) peptide (H3K4me3). Therefore, autoinhibition of MORC3 (ATPase-CW) with AMPPNP, which is disrupted by binding to H3K4me3, promotes the localization of MORC3 to H3K4me3-marked chromatin [24]. In MORC4, the ATPase and CW domains cooperate in the process of MORC4 binding to DNA, including to the nucleosome core particle (NCP), which enhances DNA wrapping around the histone core and impedes DNA-associated protein binding to the NCP [26]. Furthermore, the MORC4 ATPase activity is dependent on DNA-binding function of both the ATPase and CW domain [26], whereas the ATPase domain of MORC2 is required for human silencing hub (HUSH)-dependent silencing of transgenes integrated at chromatin loci with H3K9me3 instead of recognizing H3K4me3 [15, 27]. Similarly, the ATPase, CW and coiled-coil domains of MORC2 are required for the function of HUSH complex via dimerization upon ATP binding and dissociation upon ATP hydrolysis [15]. In addition, MORC2 forms the HUSH complex with MPP8 and TASOR, which is considered to be the most important aspect of MORC2 function. HUSH and MORC2 can selectively bind transcriptional factors and promote the trimethylation of histone H3 at Lys9 (H3K9me3) to silence transcription [15].

### Zf-CW domain

The Zinc finger (Zf)-CW domain is a relatively small motif that contains ~50–60 amino acids with four conserved cysteines (C) and two conserved tryptophans (W), which can bind zinc to form finger-like folds. This enables tandem contact with target molecules, which has been reported to be involved in chromatin remodeling [13], recognition of methylated histones [16], epigenetic regulation and early embryonic development [28]. The CW domains of MORC3 and MORC4 have been suggested to selectively bind to H3K4me in the tail of histone H3 [17, 23]. Additionally, the CW domain interacts with ATPase domain to form a complex that blocks the binding of the ATPase to DNA, which is required for the catalytic activity of MORC3 [23, 25]. Furthermore, the CW domain has been suggested to be essential in the subcellular localization of MORCs in cells, inducing the recruitment of MORC3 to the nucleus accumulating on chromatin by selectively interacting with gene promoters enriched with H3K4me3 [29–31]. In addition, an aromatic cage in the CW domains in MORC3 and MORC4 (which are absent in MORC1 and MORC2) can bind the trimethylated lysine on H3K4me3 [15, 23, 24, 27]. Moreover, the CW domain can take part in SUMOylation, which affects protein stability and ultimately leads to cell proliferation and tumorigenesis [32].

### Coiled-coil domain

The coiled-coil domain consists of two or more α-helices forming superhelical bundles [33]. Two or three coiled-coil domains at the C-terminus are present in the human MORC family, and those domains on MORC1 and MORC2 have been observed to regulate DNA-binding properties and gene expression [15]. For instance, the C-terminal coiled-coil domain regulates MORC2 dimerization, which is required in the presence of altered nucleosome stability after DNA damage and for subsequent DNA repair signaling [34]. Deletion of the coiled-coil domain disrupts the formation of MORC2 homodimers and destabilizes the histone-DNA interaction, resulting in impaired recruitment of DNA repair proteins to chromatin and decreased cell survival [34]. Similarly, MORC3 also homodimerizes through its coiled-coil domain and ATPase domain to form MORC3 nuclear matrix domains, which function as a “molecular clamp” to bind to nuclear matrix [31]. Furthermore, an additional coiled-coil motif upstream of the zinc finger-CW domain is present in MORC1 and MORC2 but not in MORC3 or MORC4 (Fig. 1). It has been reported that the coiled-coil domain overlaps within the ATPase complex, which is responsible for the interaction of MORC1 and MORC2 with chromatin [17, 27]. Therefore, the coiled-coil domain is an essential structure in regulating nucleic acid binding, protein–protein interactions, protein assembling, protein stability and molecular recognition [18–20, 35, 36].

## Distribution characteristics

### Expression distribution

Under normal physiological conditions, human MORC proteins are distributed in a cell-type- and tissue-specific manner. According to the BioGPS analysis results by Hong et al. [12], MORC1 is mainly expressed in embryonic stem cells and thymocytes; MORC2 and MORC3 are both expressed ubiquitously; and MORC4 is mainly expressed in the placenta and pituitary gland. These results show that the expression pattern of MORC family members is primarily cell-type specific (Table 2).

**Table 2:** Expression distribution and functions of MORC family.

	Expression distribution	Function	Reference
MORC1	Male germ cells	Spermatogenesis	[1, 37]
	Memory B cells, plasmablasts, bone marrow plasma cells, and myeloma cells in patients with myeloma	Possible target for cancer vaccination or prognosis	[38]
MORC2	Ubiquitous	Regulating transcription	[39]
		DDR	[40]
		Adipogenic differentiation	[41]
	Testis, ovary and brain	Unknown	[42]
	Multiple cancer cells	Transcription repression and DDR	[8]
		Promoting invasion and metastasis	[43]
		Promoting phenotype of cancer stem cells	[44]
MORC3	Ubiquitous	Transcription repression	[24, 45]
		Regulating calcium and bone homeostasis	
	Gastric cancer	SUMO-mediated transcriptional repression	[46]
	PBLs after chemotherapy	Regulating p53 activity	[47, 48]
MORC4	Placenta and testis	Spermatogenesis	[49, 50]
	Cancer	Transcription activation	

**Fig. 2:**
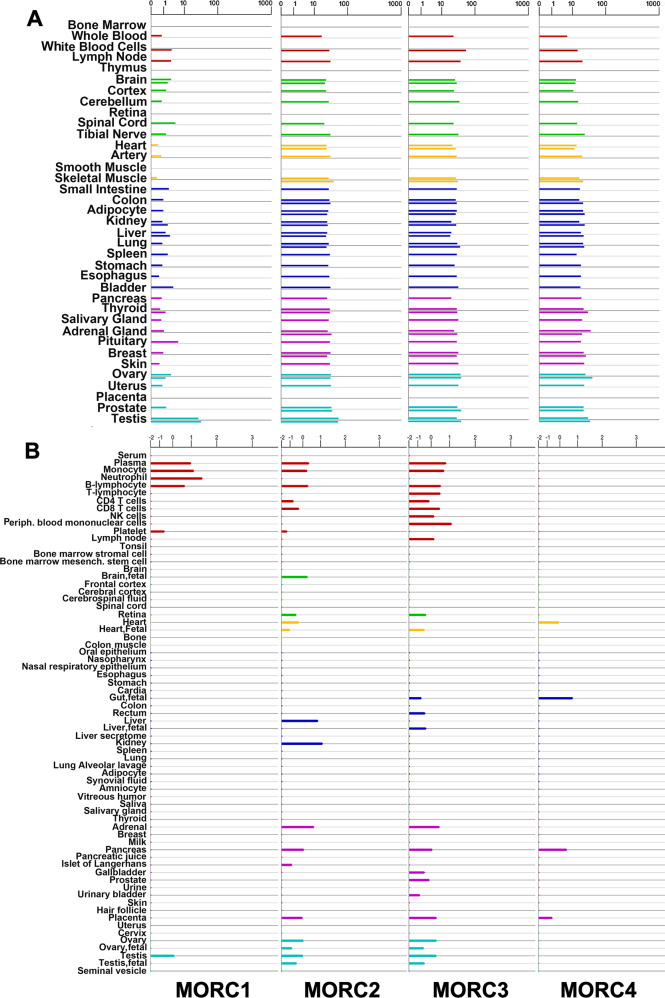
MORC family expression in normal human tissues. **A** mRNA expression of MORC family in normal tissues from GTEx; **B** protein expression in normal tissues and cell lines from Proteomics DB (https://www.genecards.org).

We obtained expression data of MORC family members through GeneCards (https://www.genecards.org). Based on the mRNA expression levels in the GTEx dataset of normal human tissues, MORC1 is distributed ubiquitously in normal human tissues, especially in reproductive tissues such as testis. MORC2, MORC3, and MORC4 are widely distributed in normal human tissues, including immune, nervous, muscle, internal, secretory and reproductive systems (Fig. 2A). However, the distribution varies considerably among normal tissues and different cell lines according to the protein expression from Proteomics DB. MORC1 protein is expressed only in the hematopoietic system (plasma, monocyte, neutrophil, and B lymphocyte) and reproductive system (testis). MORC2 protein expression is most prominent in liver and kidney, and with lower yet detectable levels in hematopoietic, immune, secretory and reproductive systems. MORC3 protein is mostly located in blood and immune, secretory and reproductive systems, also in retina, and MORC4 protein is significantly localized to heart, fetal gut, pancreas and placenta with little detected in other tissues (Fig. 2B). Thus, according to the analysis results above, MORC1-4 have specific tissue distribution. Specifically, we found differences in the mRNA and protein expression of MORC proteins in the same tissue, suggesting the global differences are due to post-transcriptional modifications.

MORC members are highly expressed in cancers and other diseases such as neurogenic disorders and metabolic bone diseases, which are related to clinicopathological parameters [23, 37, 38, 49]. This will be described in detail in the section “MORCs and Cancers and Diseases”.

### Subcellular distribution

The subcellular localization and expression of MORCs are mainly concentrated in nucleus, suggesting that MORCs are involved in transcription regulation, DNA repair/recombination, and genome stability [51]. The Protein Subcellular Localization Prediction Tool (PSORT, http://psort.hgc.jp) is a computer program for predicting protein localization sites within cells based on the amino acid sequence. According to the PSORT results (Table 3), in addition to localizing to nuclei, the MORC1 is predicted to localize mostly to the cytosol and mitochondria, with a small percentage localizing to the cytoskeleton, plasma membrane and extracellular region. The expression of MORC2 was shown to be largely confined to the nucleus under normal physiological condition, which is consistent with the PSORT results [6, 43]. However, in response to serum deprivation, MORC2 was discovered to be able to translocate from the nucleus to the cytoplasm [52]. Additionally, the PSORT results predicted that a small fraction of MORC2 resides in mitochondria and cytoskeleton. By contrast, MORC3 is highly localized to the nuclear matrix. In cells infected with influenza virus, a small fraction of MORC3 could relocate to cytoplasm and associate with the viral ribonucleoprotein complex [53, 54]. In addition to their distribution in the nucleus and cytosol, MORC3 and MORC4 are predicted to localize to mitochondria and other compartments. Therefore, based on reported evidence and predicted results, MORC family members mainly localize to the nucleus and partially localize to the cytoplasm, mitochondria and other specific subcellular compartments in response to epigenetic signals, suggesting that subcellular role of MORCs is still a largely unexplored territory.

**Table 3:** The subcellular localization of MORC family derived from PSORT database.

Compartment/Confidence	MORC1	MORC2	MORC3	MORC4
Nucleus	4	5	5	5
Cytosol	2	4	2	0
Mitochondria	2	2	2	1
Peroxisome		0		
Endoplasmic reticulm		0	1	
Cytosekelon	1	1	2	
Plasma membrane	1		1	
Extracelluar	1		1	

The confidence of each association based on sequence predictions is signified by digits, where 5 is the highest confidence and 1 is the lowest.

### MORCs and cancers

**Fig. 3:**
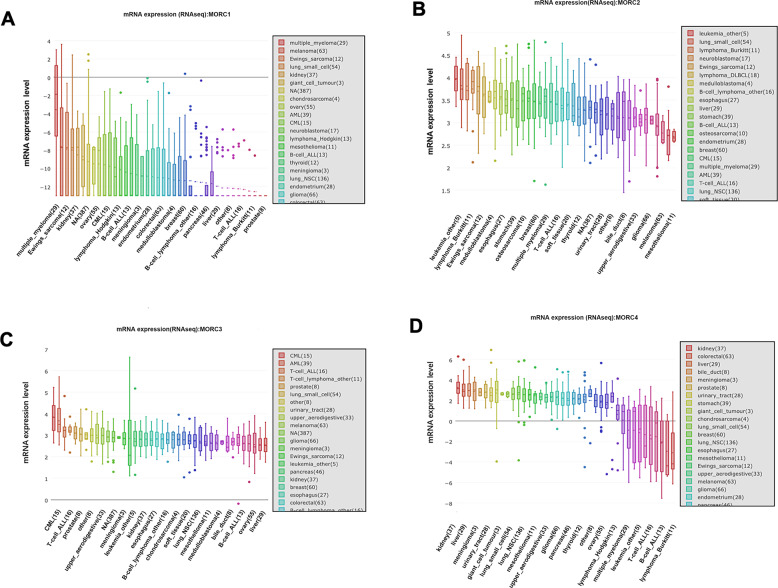
The mRNA expression of MORC family in various cancer types. The CCLE database (https://portals.broadinstitute.org/ccle/home) demonstrated the diversified expression level of MORC1 (**A**), MORC2 (**B**), MORC3 (**C**), and MORC4 (**D**) with RNA sequence databases in different cancer cell lines. MORC1 to MORC4 expressed the high level in myeloma, leukemia, breast, gastrointestinal, liver and kidney cancer cell lines. All the values of mRNA expression (RNAseq) were processed by log2-fold-change. A box plot is sorted and colored by average distribution of a gene’s expression in a lineage. Lineages are composed of a number of cell lines from the same area or system of the body. The highest average distribution is on the left and is colored red. The dashed line within a box is the mean.

**Fig. 4:**
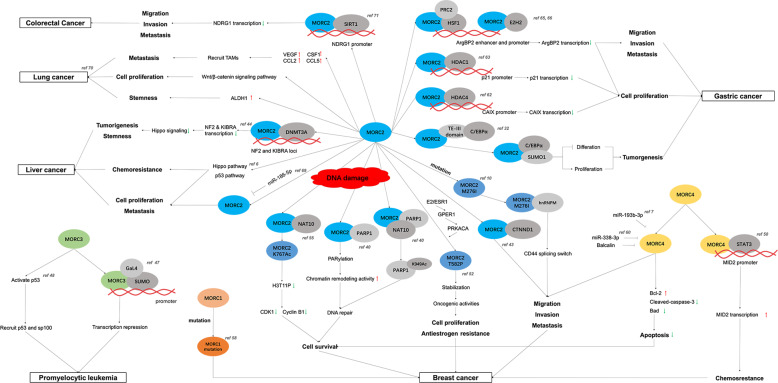
The relationship of MORC family and cancers. In regulation of DNA damage response and gene transcription, MORC family regulate the expression of key proteins by interacting with their promoters (e.g. CAIX, NDRG1, NF2, KIBRA, and MID2) or their stability/activity by direct interaction (e.g. CTNND1, C/EBPα) in pathways associated with cancer development, regulating cell proliferation, migration, invasion and metastasis of cancers, moreover affecting chemoresistance and stemness of cancers. Meanwhile, the stabilization/activity of MORC family are regulated by chromatin-associated enzymes (e.g. NAT10) and hormone (e.g. estrogen) in specific cancer, such as MORC2 in breast cancer. Furthermore, the oncogenic mutation of MORC family involves the progression of breast cancer, such as MORC1 and MORC2 M276I.

A growing number of studies have indicated that MORC proteins play a key role in the initiation and progression of cancers. According to the report of the Cancer Cell Line Encyclopedia (CCLE) project (https://portals.broadinstitute.org/ccle/home), the expression of MORC1 to MORC4 varies widely among multiple cancer types (Fig. 3). Indeed, MORC proteins are reported to regulate the DNA damage response and gene transcription, which are related to cell proliferation and survival, invasion and metastasis, chemoresistance and stemness in multiple cancers (Fig. 4). For example, MORC1 is associated with melanoma, lung cancer, and breast cancer [12]. The expression of MORC2 protein is upregulated, and MORC2 exerts its oncogenic activities in multiple cancers, including lung, prostate, liver, brain, breast, stomach, colon, pancreatic, ovarian, and endometrium cancers [8]. In addition, MORC2 expression was shown to be upregulated following endocrine therapy in breast cancer, contributing to its resistance to treatment [52], and MORC2 could regulate the resistance to chemotherapy and radiotherapy [55]. MORC3 has been reported to correlate to an increased cancer risk in patients with dermatomyositis (DM) [56]. MORC4 has been considered as a potential lymphoma biomarker due to its high expression in a subset of patients with diffuse large B-cell lymphoma [49]. Moreover, the expression level of MORC proteins in certain cancers is positively related to the degree of malignancy and is negatively related to overall survival. In addition, MORC proteins are targets of some microRNA (miRNAs) that act as important biomarkers for diagnosis, prognosis and therapy of cancer [69]. Taken together, the results indicate that MORC family members may be a promising therapeutic target.

### Breast cancer

Breast cancer, the most frequently diagnosed cancer and the leading cause of death among women worldwide, is classified into subtypes, including HER2-positive, luminal A/B and triple-negative breast cancer [57]. With the tremendous improvements in the accuracy of diagnosis and novel therapeutic agents, mortality of women with breast cancer has profoundly decreased. However, there are very few effective therapeutic methods other than surgery for treating certain subtypes of breast cancer; thus, the MORC family of proteins has emerged as potential therapeutic candidates for breast cancer treatment. Mutations of MORC have been linked to breast cancer development. A single nucleotide mutation in MORC1 was first detected in metastatic lobular breast cancer, indicating its potential role in the progression of breast cancer [58], and a mutation in MORC2 could induce the metastatic progression of triple-negative breast cancer [10].

In addition to data stating the effects of mutations on breast cancer, accumulating evidence indicates that MORC2 expression is upregulated in breast cancer and MORC2 overexpression is related to unfavorable pathological characteristics and poor prognosis. For example, MORC2 could promote the migration, invasion and metastasis of MDA-MB-231 and Hs578T cells through the interaction of its proline-rich domain with catenin delta 1 (CTNND1) [43]. MORC2 was also shown to regulate the DNA damage response through a poly (ADP-ribose) polymerase 1 (PARP1)-dependent pathway. Upon DNA damage, PARP1 recruits MORC2 to DNA damage sites and catalyzes MORC2 PARylation, which stimulates its ATPase and chromatin remodeling activities, resulting in cell survival; moreover, MORC2 stabilizes PARP1 by enhancing N-acetyltransferase 10 (NAT10)-mediated acetylation of PARP1 at lysine, which represses its ubiquitination and subsequent degradation by E3 ubiquitin ligase CHFR [40]. NAT10, which exhibits acetyltransferase activity to regulate DNA damage and cancer development and progression, is stimulated by DNA-damaging agents and is critical for G2 checkpoint arrest [59]. DNA damage induced by chemotherapeutic agents and ionizing radiation promotes the translocation of NAT10 from the nucleolus to nucleoplasm, facilitating the acetylation of MORC2 at evolutionarily conserved lysine 767 (K767Ac) and resulting in DNA damage-induced reduction in H3T11P expression and transcriptional repression of the downstream target genes CDK1 and Cyclin B1, thus resulting in DNA damage-induced G2 checkpoint activation and cell survival in breast cancer [55]. Furthermore, MORC2 is primarily degraded in the lysosome upon interacting with HSPA8 and LAMP2A through the chaperone-mediated autophagy CMA pathway. The stabilization of MORC2 is also related to breast cancer proliferation induced by estrogen and its analogs (such as 17β-estradiol) and resistance to antiestrogens (such as tamoxifen and fulvestrant) via a GPER1-PRKACA-CMA pathway [52]. Similarly, PRKACA kinase, which is activated by GPER1-mediated phosphorylation of MORC2 at T582, protects MORC2 from degradation. Then, the stabilized MORC2 exerts oncogenic functions to facilitate E2-induced cell proliferation and increase cellular resistance to antiestrogen therapies [52].

Among the members of the MORC family, MORC4 is negatively regulated by miR-193b-3p and miR-338-3p in breast cancer, suggesting the significant value of miRNAs and MORC4 in cancer treatment [7, 60]. Other reports stated that silencing MORC4 could downregulate Bcl-2 levels and upregulate cleaved-caspase-3 and Bad levels in MDA-MB-231 cells, indicating a key role of MORC4 in mediating apoptosis in breast cancer [7]. Moreover, the expression of miR-338-3p and MORC4 could be regulated by baicalin to suppress cell viability, migration and invasion and to promote apoptosis in breast cancer [60]. In addition, MORC4 recruits STAT3 to the MID2 promoter and drives MID2 expression in luminal A/B breast cancer cells, which enhances the chemoresistance of breast cancer, indicating that MORC4 may be a therapeutic target for luminal A/B breast cancer therapy [50].

### Gastric cancer

Gastric cancer is one of the most common malignancies worldwide and the leading cause of cancer-related death owing to its diagnosis at an advanced or metastatic stage due to an asymptomatic early stage [61]. Thus, to improve patient survival, it is of vital importance to understand the mechanisms and proteins that govern the development and progression of gastric cancer and screen out novel targets for diagnosing and/or treating gastric cancer. We have been dedicated in determining the exclusive role of MORC2 in gastric cancer, and we discovered its transcriptional repressor role in gastric cancer. We observed that MORC2 can bind to target promoters and repress their transcription, which is crucial in cell proliferation and survival regulation, eventually leading to gastric cancer progression. For instance, MORC2 binds to carbonic anhydrase IX (CAIX) and recruits histone deacetylase 4 (HDAC4), which deacetylates of histone H3 at the promoter and represses the transcription of the CAIX gene; this gene plays a key role in gastric tumor cell growth and survival [39]. Then, we found that the phosphorylation of MORC2 at Ser-677 (MORC2-S677A) by p21-activated kinase 1 (PAK1) promotes gastric cancer cell proliferation and tumorigenesis. The correlation of MORC2 and the PAK1 pathway has also demonstrated in clinical studies showing that higher levels of PAK1 expression and phosphorylated MORC2 correlates with shorter overall survival and poor prognosis of clinical gastric cancer [62]. We also found that MORC2 could mediate the downregulation of p21 by recruiting HDAX1 to the p21 promoter independent of p53 status, leading to cell proliferation in MORC2/SGC-7901 cells (a gastric cancer cell line with stable exogenous expression of MORC2) [63]. Moreover, it has been reported that Arg kinase-binding protein 2 (ArgBP2) is absent in many kinds of malignant metastatic cancers [64] and is an adapter protein that correlates with the proliferation, adhesion, and migration of gastric tumors [65]. Likewise, MORC2 in association with heat shock factor (HSF1) facilitates the recruitment of polycomb repressive complex 2, which in particular promotes the recruitment of enhancer of zeste homolog 2 and the consequent trimethylation of lysine 27 on histone H3 (H3K27me3), resulting in the repression of the ArgBP2 transcription in human gastric adenocarcinoma cell lines SGC-7901 and BGC-823 [65, 66]. In addition, CCAAT/enhancer-binding protein α (C/EBPα) is one type of transcription factor that can control cellular proliferation and differentiation in the gastric cancer cell lines MKN45 and MKN74 [67]. We found that MORC2 promotes the SUMOylation and subsequent degradation of C/EBPα by interacting with TE-III domain of C/EBPα, contributing to proliferation of SGC-7901 and BGC-823 cells; meanwhile, MORC2 represses C/EBPα-mediated cell differentiation of mouse C2C12 cells, which is essential for maintaining cell cycle progression [32]. Remarkably, high levels of MORC2 expression are correlated with an aggressive phenotype of clinical gastric cancer and shorter overall survival of patients [32]. These findings all suggest MORC2 as a potential therapeutic target in gastric cancer. However, other MORC members have not been reported to be associated with gastric cancer.

### Liver cancer

Primary liver cancer, including hepatocellular carcinoma (HCC) and cholangiocarcinoma (CCA), is a prevalent malignancy worldwide in which HCC accounts for 70–80% cases and CCA accounts for 15% [68]. Upregulated MORC2 expression has been observed in HCC tissues and is associated with the clinicopathological features of patients with HCC. MORC2 regulates the intrinsic apoptotic pathway via p53 and Hippo, thus contributing to the proliferation, metastasis, and chemoresistance of liver cancer cell lines, including HepG2, Bel-7402, Huh7, PLC/PRF-5, SMMC7721, and LM3. The proliferation and clonogenicity of liver cancer cells, as well as their migratory and invasive abilities, could be inhibited by knocking down MORC2 [6]. MORC2 binds the DNA methyltransferase DNMT3A and facilitates DNA hypermethylation, resulting in transcriptional repression at neurofibromatosis 2 (NF2) and kidney and brain protein (KIBRA), which are upstream activators of Hippo signaling pathway. This plays a key role in promoting HCC stemness, chemoresistance, and tumorigenesis [44]. In addition, in the CCA cell lines QBC-939 and RBE, MORC2 is a target of miR-186-5p, which represses cell proliferation, migration, and metastasis upon negatively regulating MORC2 [69]. However, the precise mechanism of dysregulation of MORC2 in liver cancer is still unclear.

### Other cancers

In addition to the cancer types described above, there are clinical and prognostic correlations of MORC members with other cancers. For instance, in promyelocytic leukemia (PML), MORC3 interacts with small ubiquitin-like modifier (SUMO) to target a Gal4-dependent promoter, resulting in SUMO-mediated transcriptional repression [46]. MORC3 also regulates the activity of the tumor suppressor p53, which is a key transcriptional factor regulating the induction of cellular senescence via oncogenic signals in PML [47]. In addition, MORC2 has been reported to play a role in lung and colorectal cancer (CRC) in recent years. MORC2 could activate the Wnt/β-catenin signaling pathway and stimulate the recruitment of tumor-associated macrophages, contributing to the promotion of lung tumor growth and metastasis in a series of lung cancer cell lines, including A549, PC-9, H1299, NCI-H2170, NCI-H226, and LL/2 cells [70]. Furthermore, MORC2 increases the level of aldehyde dehydrogenase-1 protein in lung cancer, which is regarded as a feature of cancer stem cells [70]. In CRC cell lines (HT29, SW-480, and SW620), MORC2 can downregulate the expression of NDRG1, a metastasis suppressor and prognostic biomarker of CRC, by interacting with sirtuin 1 and binding to the NDRG1 promoter. Moreover, MORC2 mediates increases in CRC cell migration and invasion in vitro and promotes lung metastasis of CRC cells in vivo, indicating that MORC2 is a potential therapeutic target for CRC [71]. However, the high level of serum MORC2 protein was suggested to correlate with a favorable prognosis in CRC based on an analysis of plasma samples from 76 CRC patients via UPLC-UDMS^E^ based proteomics [72]. Further study on the relationship between the MORC family and other cancers will be of great importance in understanding the mechanism by which they contribute to cancer development and their potential roles in cancer therapy.

### MORCs and other diseases

The overexpression of MORC proteins is not unique to cancer. Mutations of MORC proteins are often observed in cancers, for example, the substitution mutation of MORC2 from methionine to isoleucine at residue 276 (M276I) promoted cell migration, invasion, and lung metastasis of breast cancer [10]. However, MORC mutant proteins are also found in other diseases, such as neurogenic disorders and metabolic bone diseases.

**Fig. 5:**
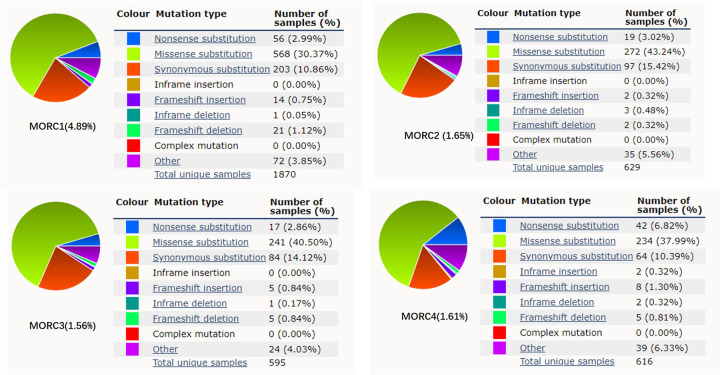
An overview of the types of MORC family mutation observed. According to the Cosmic public databases (https://cancer.sanger.ac.uk/cosmic), these charts show a summary of the types of mutation that have been observed in samples for MORCs gene. These tables show the number of samples recorded as having a particular type of mutation, with the number in brackets giving the percentage of samples with that type of mutation. Meanwhile, note that a sample may have more than one type of mutation, so the total number of samples determined by simply summing the values in the table may not match the total number of unique samples given under the table. For the same reason, summing the percentages in the table may give a value of greater than 100%.

To obtain a more global understanding of the types of mutations in MORC proteins, we explored Cosmic public databases (https://cancer.sanger.ac.uk/cosmic) and found that relevant MORC mutations have been identified. As shown in Fig. 5, the mutation rate of human MORC1-4 was 4.89%, 1.65%, 1.56%, and 1.61%, respectively. There were 1870 unique samples with observed MORC1 mutations in 38237 samples; 629 unique samples with mutated MORC2 in 38171 samples; 595 unique samples with MORC3 mutations in 38173 samples and 616 unique samples with MORC4 mutations in 38170 samples. A variety of mutations were observed, primarily missense substitutions (> 30%) followed by synonymous substitutions (>10%). Therefore, the aberrant expression or mutation of MORC members is involved not only in some cancers but also in several diseases, including inflammation and immunopathologies, bone diseases and other disorders (Table 4).

**Table 4:** MORC family associated diseases.

MORC members	Associated diseases	Cell lines	Disease models or patients	Reference
MORC1	Male infertility	–	Men with infertility	[73]
	Major depressive disorder (MDD)	CD34+ cells from human cord blood CD3+ T cells from monkey peripheral blood	Prefrontal cortex of adult rats	[74, 75]
MORC2	CMT	Hela cells, patient-derived fibroblasts, and primary neuronal cultures	CMT2 patients	[76, 77]
MORC3	Metabolic bone diseases	–	MORC^3+/−^ mice	[45]
	Virus infection	–	Herpes simplex virus 1; human cytomegalovirus	[78]
	Dermatomyositis	–	Dermatomyositis patients	[79]
	Down syndrome	D21 and T21 fibroblasts, D21 and T21 lymphoblastoid cells	Mouse Ts1Cje model of DS	[23]
	Inflammatory disease	–	Patients with IIMs	[80]
MORC4	Diffuse large B-cell lymphomas	GC- and NGC- DLBCL cell lines	Patients with DLBCL	[49]
	Inflammatory disease	–	Patients with pancreatitis	[81]

### Inflammation and immunopathologies

MORC members, such as MORC3 (which is abundant in immune cells), have been reported to be associated with certain inflammatory conditions and immunopathologies [12, 82]. For example, anti-MORC3 antibody has been confirmed as one of the major myositis-specific autoantibodies, which are essential in identifying clinically homogeneous subsets and predicting the prognosis of idiopathic inflammatory myopathies (IIMs) (such as polymyositis and DM) [83]. Among them, DM is an autoimmune disease associated with specific autoantibodies, which are closely correlated with distinct clinical manifestations. DM-specific autoantibodies include antibodies against melanoma differentiation antigen 5, transcriptional intermediary factor 1, antinuclear matrix protein NXP2 (MORC3), and SUMO activating enzyme [84]. Significant muscle ischemia and anti-MORC3 autoantibodies were observed in association with severe forms of juvenile DM [85]. MORC3 also serves as a useful marker for disease activity in patients with IIMs, especially in the absence of calcinosis [80]. Patients administered anti-MORC3 antibodies had a lower rate of remission than those who did not receive this treatment [86]. In a case report of immune-mediated necrotizing myopathy (IMNM) associated with acute myeloid leukemia, anti-MORC3 antibody was detected during active myositis but disappeared after disease remission, indicating that anti-MORC antibody could be used as a diagnostic and prognostic marker of paraneoplastic IMNMs [87]. Anti-MORC3 antibody is also associated with abdominal pathologies with several manifestations, such as intestinal vasculitis, ulcers, bleeding and dysphagia [79]. However, the precise mechanism of MORC3 with regard to inflammation and immunopathologies is unclear. MORC4 has been implicated in several types of inflammatory diseases, such as Crohn’s disease, ulcerative colitis, tropical calcific pancreatitis, and acute/chronic pancreatitis [81, 88–90]. In several genome-wide association studies, the association of variants in claudin2 (CLDN2), MORC4 and PRSS1-PRSS2 loci with pancreatitis have identified in certain populations [88, 90]. However, MORC4 and CLDN2 have been specifically indicated as having a significant association with chronic pancreatitis, in which the downstream signaling of MORC4 activation might be interact with proteins known to be associated with chronic pancreatitis, including AMPKa1, GEMIN4, HECW2, SKIL, STAT3, and UBC [90].

### Bone diseases

In addition to being regarded as an antigen for DM, which is associated with the skin calcinosis (calcium deposition under the skin), MORC3 has been shown to regulate cortical bone homeostasis and the hematopoietic stem cell niche [45, 91]. MORC3-mutant mice displayed reduced cortical area and thickness with increased cortical porosity. Some mutations in MORC3 activate the STAT1 signaling pathway and alter Stat1 and Ifnb1 gene expression, resulting in an increase in osteoblast differentiation and changes in osteoblastic gene expression [45]. Therefore, MORC3 is also a transcriptional regulator of proteins involved in calcium and bone homeostasis.

### Other disorders

MORC proteins are also related to neuropathies in humans. Through a gene-set-based analysis of data from a genome-wide association study of major depressive disorder (MDD), the association of MORC1 with MDD was revealed, that is, MORC1 was suggested as a gene involved in the immediate response to early life stress [74]. It was found that MORC1 is differentially methylated in the brain and peripheral cells at different ages, which could be followed longitudinally in living humans [74]. Additionally, MORC2 is associated with the activity of HDAC4, which is also an important protein in synaptic plasticity and regulates transcription in the central nervous system [39]. The mutations in MORC2 have been reported to be causally associated with several neuropathic disorders, such as Charcot-Marie-Tooth (CMT) disease, cerebellar ataxia, axonal polyneuropathy, and nocturnal hypoventilation [77, 92, 93]. Pathogenic variants in the ATPase domain of MORC2 has been suggested to cause hyperactivation of epigenetic silencing by the HUSH complex, which contributes to neurodevelopmental disorders with growth retardation and variable craniofacial dysmorphism [94]. In addition, MORC3 is reported to regulate influenza virus infection. The downregulation of MORC3 represses virus titers and the accumulation of viral genomic RNA and mRNAs, and silencing MORC3 reduces CAN mRNA and protein levels in the influenza virus CAT minireplicon system, indicating the role of MORC3 in influenza virus transcription [54]. MORC3 expression is also markedly upregulated in individuals with Down Syndrome (DS), indicating its correlation with DS compared to that of MORC2 and MORC4 [23].

## Conclusions

Mounting evidence has revealed the diverse functions of the MORC protein family based on their unique structural characteristics and distribution patterns. MORC proteins play an important role in DNA damage repair and transcriptional repression, thus controlling the cancer cell cycle, and have been regarded as crucial proteins responsible for gene silencing and disease progression, including regulating cancer occurrence and development and modulating immune system and skeletal system. These findings are only the tip of the iceberg, as more characteristics and biological functions for MORC proteins are still awaiting discovery. Particularly, insights into how MORC proteins modulate cancer progression remain unclear. This review takes advantage of the rapidly increasing understanding of the roles of MORCs in cancer and disease.

Although the functions of MORCs are beginning to emerge, most are still uncharacterized. For example, the functions of mutations and the subcellular localization of MORCs, to the best of our knowledge, are unknown. Future studies that elucidate the unknown function and regulation of these MORCs will expand our understanding of MORCs as a focal point for diverse disease progression and related signaling. Thus, advanced knowledge of the MORC protein family will be of great value in establishing MORCs as new targets in the diagnosis and treatment for multiple cancers and disease disorders.

## Supplementary information

author-contribution-form-

## Data Availability

All data generated during and/or analyzed during the current study are available.

## References

[1] Inoue N, Hess KD, Moreadith RW, Richardson LL, Handel MA, Watson ML (1999). New gene family defined by MORC, a nuclear protein required for mouse spermatogenesis. Hum Mol Genet.

[2] Koch A, Kang H-G, Steinbrenner J, Dempsey DMA, Klessig DF, Kogel K-H (2017). MORC proteins: novel players in plant and animal health. Front Plant Sci.

[3] Li D-Q, Nair SS, Kumar R (2014). The MORC family. Epigenetics.

[4] Perry J, Zhao Y (2003). The CW domain, a structural module shared amongst vertebrates, vertebrate-infecting parasites and higher plants. Trends Biochem Sci.

[5] Dong W, Vannozzi A, Chen F, Hu Y, Chen Z, Zhang L (2018). MORC domain definition and evolutionary analysis of the MORC gene family in green plants. Genome Biol Evol.

[6] Pan Z, Ding Q, Guo Q, Guo Y, Wu L, Wu L (2018). MORC2, a novel oncogene, is upregulated in liver cancer and contributes to proliferation, metastasis and chemoresistance. Int J Oncol.

[7] Yang ZA, Zhuang Q, Hu G (2018). is a novel breast cancer oncogene regulated by miR‐193b‐3p. J Cell Biochem.

[8] Ding QS, Zhang L, Wang BC, Zeng Z, Zou XQ, Cao PB (2018). Aberrant high expression level of MORC2 is a common character in multiple cancers. Hum Pathol.

[9] Sevilla T, Lupo V, Martínez-Rubio D, Sancho P, Sivera R, Chumillas MJ (2016). Mutations in the MORC2 gene cause axonal Charcot–Marie–Tooth disease. Brain.

[10] Zhang FL, Cao JL, Xie HY, Sun R, Yang LF, Shao ZM (2018). Cancer-associated MORC2-mutant M276I regulates an hnRNPM-mediated CD44 splicing switch to promote invasion and metastasis in triple-negative breast cancer. Cancer Res.

[11] Liu ZW, Zhou JX, Huang HW, Li YQ, Shao CR, Li L (2016). Two Components of the RNA-directed DNA methylation pathway associate with MORC6 and silence loci targeted by MORC6 in arabidopsis. PLoS Genet.

[12] Hong G, Qiu H, Wang C, Jadhav G, Wang H, Tickner J (2017). The emerging role of MORC family proteins in cancer development and bone homeostasis. J Cell Physiol.

[13] Li DQ, Nair SS, Ohshiro K, Kumar A, Nair VS, Pakala SB (2012). MORC2 signaling integrates phosphorylation-dependent, ATPase-coupled chromatin remodeling during the DNA damage response. Cell Rep.

[14] Kim H, Yen L, Wongpalee SP, Kirshner JA, Mehta N, Xue Y (2019). The gene-silencing protein MORC-1 topologically entraps DNA and forms multimeric assemblies to cause DNA compaction. Mol Cell.

[15] Douse CH, Bloor S, Liu Y, Shamin M, Tchasovnikarova IA, Timms RT (2018). Neuropathic MORC2 mutations perturb GHKL ATPase dimerization dynamics and epigenetic silencing by multiple structural mechanisms. Nat Commun.

[16] Dobrovolska O, Brilkov M, Madeleine N, Odegard-Fougner O, Stromland O, Martin SR (2020). The Arabidopsis (ASHH2) CW domain binds monomethylated K4 of the histone H3 tail through conformational selection. FEBS J.

[17] Liu Y, Tempel W, Zhang Q, Liang X, Loppnau P, Qin S (2016). Family-wide characterization of histone binding abilities of human CW domain-containing proteins. J Biol Chem.

[18] Li X, He L, Che KH, Funderburk SF, Pan L, Pan N (2012). Imperfect interface of Beclin1 coiled-coil domain regulates homodimer and heterodimer formation with Atg14L and UVRAG. Nat Commun.

[19] Matityahu A, Onn I (2017). A new twist in the coil: functions of the coiled-coil domain of structural maintenance of chromosome (SMC) proteins. Curr Genet.

[20] Terawaki S-i, Yoshikane A, Higuchi Y, Wakamatsu K (2015). Structural basis for cargo binding and autoinhibition of Bicaudal-D1 by a parallel coiled-coil with homotypic registry. Biochem Biophys Res Commun.

[21] Pastor WA, Stroud H, Nee K, Liu W, Pezic D, Manakov S (2014). MORC1 represses transposable elements in the mouse male germline. Nat Commun.

[22] Iyer LM, Abhiman S, Aravind L (2008). MutL homologs in restriction-modification systems and the origin of eukaryotic MORC ATPases. Biol Direct.

[23] Andrews FH, Tong Q, Sullivan KD, Cornett EM, Zhang Y, Ali M (2016). Multivalent chromatin engagement and inter-domain crosstalk regulate MORC3 ATPase. Cell Rep.

[24] Li S, Yen L, Pastor WA, Johnston JB, Du J, Shew CJ (2016). Mouse MORC3 is a GHKL ATPase that localizes to H3K4me3 marked chromatin. Proc Natl Acad Sci USA.

[25] Zhang Y, Klein BJ, Cox KL, Bertulat B, Tencer AH, Holden MR (2019). Mechanism for autoinhibition and activation of the MORC3 ATPase. Proc Natl Acad Sci USA.

[26] Tencer AH, Cox KL, Wright GM, Zhang Y, Petell CJ, Klein BJ (2020). Molecular mechanism of the MORC4 ATPase activation. Nat Commun.

[27] Tchasovnikarova IA, Timms RT, Douse CH, Roberts RC, Dougan G, Kingston RE (2017). Hyperactivation of HUSH complex function by Charcot–Marie–Tooth disease mutation in MORC2. Nat Genet.

[28] Liu Y, Liu S, Zhang X, Liang X, Zahid KR, Liu K (2016). Structure and function of CW domain containing proteins. Curr Protein Pept Sci.

[29] He F, Umehara T, Saito K, Harada T, Watanabe S, Yabuki T (2010). Structural insight into the zinc finger CW domain as a histone modification reader. Structure.

[30] Zhang Y, Bertulat B, Tencer AH, Ren X, Wright GM, Black J (2019). MORC3 forms nuclear condensates through phase separation. iScience.

[31] Mimura Y, Takahashi K, Kawata K, Akazawa T, Inoue N (2010). Two-step colocalization of MORC3 with PML nuclear bodies. J Cell Sci.

[32] Liu J, Zhang Q, Ruan B, Chen W, Zheng J, Xu B (2019). MORC2 regulates C/EBPα-mediated cell differentiation via sumoylation. Cell Death Differ.

[33] Lupas AN, Bassler J, Dunin-Horkawicz S (2017). The structure and topology of α-helical coiled coils. Subcell Biochem.

[34] Xie HY, Zhang TM, Hu SY, Shao ZM, Li DQ (2019). Dimerization of MORC2 through its C-terminal coiled-coil domain enhances chromatin dynamics and promotes DNA repair. Cell Commun Signal.

[35] Yoshinaka T, Kosako H, Yoshizumi T, Furukawa R, Hirano Y, Kuge O (2019). Structural basis of mitochondrial scaffolds by prohibitin complexes: insight into a role of the coiled-coil region. iScience.

[36] Cristie-David AjithaS, Chen Junjie, Nowak DerekB, Bondy AmyL, Sun Kai, Park SungI (2019). Coiled-coil-mediated assembly of an icosahedral protein cage with extremely high thermal and chemical stability. J Am Chem Soc.

[37] Weiser NE, Yang DX, Feng S, Kalinava N, Brown KC, Khanikar J (2017). MORC-1 integrates nuclear RNAi and transgenerational chromatin architecture to promote germline immortality. Dev Cell.

[38] Condomines M, Hose D, Raynaud P, Hundemer M, De Vos J, Baudard M (2007). Cancer/testis genes in multiple myeloma: expression patterns and prognosis value determined by microarray analysis. J Immunol.

[39] Shao Y, Li Y, Zhang J, Liu D, Liu F, Zhao Y (2010). Involvement of histone deacetylation in MORC2-mediated down-regulation of carbonic anhydrase IX. Nucleic Acids Res.

[40] Zhang L, Li DQ (2019). MORC2 regulates DNA damage response through a PARP1-dependent pathway. Nucleic Acids Res.

[41] Sánchez-Solana B, Li D-Q, Kumar R (2014). Cytosolic functions of MORC2 in lipogenesis and adipogenesis. Biochim Biophys Acta.

[42] Nagase T, Ishikawa K, Suyama M, Kikuno R, Hirosawa M, Miyajima N (1998). Prediction of the coding sequences of unidentified human genes. XII. The complete sequences of 100 new cDNA clones from brain which code for large proteins in vitro. DNA Res.

[43] Liao XH, Zhang Y, Dong WJ, Shao ZM, Li DQ (2017). Chromatin remodeling protein MORC2 promotes breast cancer invasion and metastasis through a PRD domain-mediated interaction with CTNND1. Oncotarget.

[44] Wang T, Qin ZY, Wen LZ, Guo Y, Liu Q, Lei ZJ (2018). Epigenetic restriction of Hippo signaling by MORC2 underlies stemness of hepatocellular carcinoma cells. Cell Death Differ.

[45] Jadhav G, Teguh D, Kenny J, Tickner J, Xu J (2016). Morc3 mutant mice exhibit reduced cortical area and thickness, accompanied by altered haematopoietic stem cells niche and bone cell differentiation. Sci Rep.

[46] Rosendorff A, Sakakibara S, Lu S, Kieff E, Xuan Y, DiBacco A (2006). NXP-2 association with SUMO-2 depends on lysines required for transcriptional repression. Proc Natl Acad Sci USA.

[47] Takahashi K, Yoshida N, Murakami N, Kawata K, Ishizaki H, Tanaka-Okamoto M (2007). Dynamic regulation of p53 subnuclear localization and senescence by MORC3. Mol Biol Cell.

[48] Gonzalez-Fernandez R, Morales M, Avila J, Martin-Vasallo P (2012). Changes in leukocyte gene expression profiles induced by antineoplastic chemotherapy. Oncol Lett.

[49] Liggins AP, Cooper CDO, Lawrie CH, Brown PJ, Collins GP, Hatton CS (2007). MORC4, a novel member of the MORC family, is highly expressed in a subset of diffuse large B-cell lymphomas. Br J Haematol.

[50] Luo J, Zeng S, Tian C (2020). MORC4 promotes chemoresistance of luminal A/B breast cancer via STAT3-mediated MID2 upregulation. Onco Targets Ther.

[51] Wang G-L, Wang C-Y, Cai X-Z, Chen W, Wang X-H, Li F (2010). Identification and expression analysis of a novel CW-type zinc finger Protein MORC2 in cancer cells. Anat Rec.

[52] Yang F, Xie H-Y, Yang L-F, Zhang L, Zhang F-L, Liu H-Y (2019). Stabilization of MORC2 by estrogen and antiestrogens through GPER1- PRKACA-CMA pathway contributes to estrogen-induced proliferation and endocrine resistance of breast cancer cells. Autophagy.

[53] Kimura Y, Sakai F, Nakano O, Kisaki O, Sugimoto H, Sawamura T (2002). The newly identified human nuclear protein NXP-2 possesses three distinct domains, the nuclear matrix-binding, RNA-binding, and coiled-coil domains. J Biol Chem.

[54] Ver LS, Marcos-Villar L, Landeras-Bueno S, Nieto A, Ortin J (2015). The cellular factor NXP2/MORC3 Is a positive regulator of influenza virus multiplication. J Virol.

[55] Liu HY, Liu YY, Yang F, Zhang L, Zhang FL, Hu X (2020). Acetylation of MORC2 by NAT10 regulates cell-cycle checkpoint control and resistance to DNA-damaging chemotherapy and radiotherapy in breast cancer. Nucleic Acids Res.

[56] Fiorentino DF, Chung LS, Christopher-Stine L, Zaba L, Li S, Mammen AL (2013). Most patients with cancer-associated dermatomyositis have antibodies to nuclear matrix protein NXP-2 or transcription intermediary factor 1gamma. Arthritis Rheum.

[57] Torre LA, Islami F, Siegel RL, Ward EM, Jemal A (2017). Global cancer in women: burden and trends. Cancer Epidemiol Biomark Prev.

[58] Shah SP, Morin RD, Khattra J, Prentice L, Pugh T, Burleigh A (2009). Mutational evolution in a lobular breast tumour profiled at single nucleotide resolution. Nature.

[59] Liu X, Tan Y, Zhang C, Zhang Y, Zhang L, Ren P (2016). NAT10 regulates p53 activation through acetylating p53 at K120 and ubiquitinating Mdm2. EMBO Rep.

[60] Duan X, Guo G, Pei X, Wang X, Li L, Xiong Y (2019). Baicalin inhibits cell viability, migration and invasion in breast cancer by regulating miR-338-3p and MORC4. Onco Targets Ther.

[61] Johnston FM, Beckman M (2019). Updates on management of gastric cancer. Curr Oncol Rep.

[62] Wang G, Song Y, Liu T, Wang C, Zhang Q, Liu F (2015). PAK1-mediated MORC2 phosphorylation promotes gastric tumorigenesis. Oncotarget.

[63] Zhang Q, Song Y, Chen W, Wang X, Miao Z, Cao L (2015). By recruiting HDAC1, MORC2 suppresses p21Waf1:Cip1 in gastric cancer. Oncotarget.

[64] Anekal PV, Yong J, Manser E (2015). Arg kinase-binding protein 2 (ArgBP2) interaction with alpha-actinin and actin stress fibers inhibits cell migration. J Biol Chem.

[65] Tong Y, Li Y, Gu H, Wang C, Liu F, Shao Y (2015). Microchidia protein 2, MORC2, downregulates the cytoskeleton adapter protein, ArgBP2, via histone methylation in gastric cancer cells. Biochem Biophys Res Commun.

[66] Tong Y, Li Y, Gu H, Wang C, Liu F, Shao Y (2018). HSF1, in association with MORC2, downregulates ArgBP2 via the PRC2 family in gastric cancer cells. Biochim Biophys Acta Mol Basis Dis.

[67] Tomizawa M, Shinozaki F, Motoyoshi Y, Sugiyama T, Yamamoto S, Ishige N (2017). CCAAT/enhancer-binding protein α decreases the viability of gastric cancer cells. Oncol Lett.

[68] Kim S, Yang Y, Seki E (2019). Inflammation and liver cancer: molecular mechanisms and therapeutic targets. Semin Liver Dis.

[69] Liao G, Liu X, Wu D, Duan F, Xie X, Wen S (2019). MORC2 promotes cell growth and metastasis in human cholangio- carcinoma and is negatively regulated by miR-186-5p. Aging.

[70] Liu M, Sun X, Shi S (2018). MORC2 enhances tumor growth by promoting angiogenesis and tumor-associated macrophage recruitment via Wnt/beta-catenin in lung cancer. Cell Physiol Biochem.

[71] Liu J, Shao Y, He Y, Ning K, Cui X, Liu F (2019). MORC2 promotes development of an aggressive colorectal cancer phenotype through inhibition of NDRG1. Cancer Sci.

[72] Holm M, Joenvaara S, Saraswat M, Mustonen H, Tohmola T, Ristimaki A (2019). Identification of several plasma proteins whose levels in colorectal cancer patients differ depending on outcome. FASEB Bioadv.

[73] Cheung S, Parrella A, Rosenwaks Z, Palermo GD (2019). Genetic and epigenetic profiling of the infertile male. PLoS One.

[74] Nieratschker V, Massart R, Gilles M, Luoni A, Suderman MJ, Krumm B (2014). MORC1 exhibits cross-species differential methylation in association with early life stress as well as genome-wide association with MDD. Transl Psychiatry.

[75] Schmidt M, Brandwein C, Luoni A, Sandrini P, Calzoni T, Deuschle M (2016). Morc1 knockout evokes a depression-like phenotype in mice. Behav Brain Res.

[76] Sevilla T, Lupo V, Martinez-Rubio D, Sancho P, Sivera R, Chumillas MJ (2016). Mutations in the MORC2 gene cause axonal Charcot-Marie-Tooth disease. Brain.

[77] Sancho P, Bartesaghi L, Miossec O, Garcia-Garcia F, Ramirez-Jimenez L, Siddell A (2019). Characterization of molecular mechanisms underlying the axonal Charcot-Marie-Tooth neuropathy caused by MORC2 mutations. Hum Mol Genet.

[78] Sloan E, Orr A, Everett RD (2016). MORC3, a component of PML nuclear bodies, has a role in restricting herpes simplex viurs 1 and human cytomegalovirus. J Virol.

[79] Rogers A, Chung L, Li S, Casciola-Rosen L, Fiorentino DF (2017). Cutaneous and systemic findings associated with nuclear matrix protein 2 antibodies in adult dermatomyositis patients. Arthritis Care Res.

[80] Yang H, Lu X, Peng Q, Jiang W, Shi J, Zhang Y (2018). Differential clinical associations of anti-nuclear matrix protein 2 autoantibodies in patients with idiopathic inflammatory myopathies. Arthritis Rheumatol.

[81] Deng Y, Li Z (2020). Effects of PRSS1-PRSS2 rs10273639, CLDN2 rs7057398 and MORC4 rs12688220 polymorphisms on individual susceptibility to pancreatitis: A meta-analysis. Genomics.

[82] Nagase T, Seki N, Tanaka A, Ishikawa K, Nomura N (1995). Prediction of the coding sequences of unidentified human genes. IV. The coding sequences of 40 new genes (KIAA0121-KIAA0160) deduced by analysis of cDNA clones from human cell line KG-1. DNA Res.

[83] Ichimura Y, Matsushita T, Hamaguchi Y, Kaji K, Hasegawa M, Tanino Y (2012). Anti-NXP2 autoantibodies in adult patients with idiopathic inflammatory myopathies: possible association with malignancy. Ann Rheum Dis.

[84] Fujimoto M, Watanabe R, Ishitsuka Y, Okiyama N (2016). Recent advances in dermatomyositis-specific autoantibodies. Curr Opin Rheumatol.

[85] Aouizerate J, De Antonio M, Bader-Meunier B, Barnerias C, Bodemer C, Isapof A (2018). Muscle ischaemia associated with NXP2 autoantibodies: a severe subtype of juvenile dermatomyositis. Rheumatology.

[86] Onuora S (2018). Myositis: Anti-NXP2 antibodies associated with severe JDM. Nat Rev Rheumatol.

[87] Su L, Yang Y, Jia Y, Liu X, Zhang W, Yuan Y (2018). Anti-NXP2-antibody-positive immune-mediated necrotizing myopathy associated with acute myeloid leukemia: a case report. Medicine.

[88] Soderman J, Noren E, Christiansson M, Bragde H, Thiebaut R, Hugot JP (2013). Analysis of single nucleotide polymorphisms in the region of CLDN2-MORC4 in relation to inflammatory bowel disease. World J Gastroenterol.

[89] Paliwal S, Bhaskar S, Nageshwar Reddy D, Rao GV, Thomas V, Singh SP (2016). Association analysis of PRSS1-PRSS2 and CLDN2-MORC4 variants in nonalcoholic chronic pancreatitis using tropical calcific pancreatitis as model. Pancreas.

[90] Giri AK, Midha S, Banerjee P, Agrawal A, Mehdi SJ, Dhingra R (2016). Common variants in CLDN2 and MORC4 genes confer disease susceptibility in patients with chronic pancreatitis. PLoS One.

[91] Gunawardena H, Wedderburn LR, Chinoy H, Betteridge ZE, North J, Ollier WE (2009). Autoantibodies to a 140-kd protein in juvenile dermatomyositis are associated with calcinosis. Arthritis Rheum.

[92] Schottmann G, Wagner C, Seifert F, Stenzel W, Schuelke M (2016). MORC2 mutation causes severe spinal muscular atrophy-phenotype, cerebellar atrophy, and diaphragmatic paralysis. Brain.

[93] Ando M, Okamoto Y, Yoshimura A, Yuan JH, Hiramatsu Y, Higuchi Y (2017). Clinical and mutational spectrum of Charcot-Marie-Tooth disease type 2Z caused by MORC2 variants in Japan. Eur J Neurol.

[94] Guillen Sacoto MJ, Tchasovnikarova IA, Torti E, Forster C, Andrew EH, Anselm I (2020). De Novo Variants in the ATPase Module of MORC2 Cause a Neurodevelopmental Disorder with Growth Retardation and Variable Craniofacial Dysmorphism. Am J Hum Genet.

