# Supplementary material for: MORC protein family-related signature within human disease and cancer
Source: Cell Death Dis. 2021 Nov 27;12(12):1112. doi: 10.1038/s41419-021-04393-1 (PMC8627505; doi:10.1038/s41419-021-04393-1)
Supplement: Supplementary file 1 — author-contribution-form- [file 41419_2021_4393_MOESM1_ESM.pdf]

DECLARATION OF CONTRIBUTIONS TO ARTICLE

ADMC

Manuscript Number:	Journal Name:	
CDDIS-21-0253R	*Cell Death & Disease*	(the 'Journal')

Proposed Title of the Contribution:

MORC family-related signature within human disease and cancer (the 'Contribution)

Author(s):

Huan Wang, Ling Zhang, Qiuhua Luo and Guiling Wang (the 'Authors')

For all *CDDis* articles, each person named as an author in the published version must be able to show he or she has contributed substantially to the article.

Authorship credit should be based on 1) substantial contributions to conception and design, acquisition of data, or analysis and interpretation of data; 2) drafting the article or revising it critically for important intellectual content; and 3) final approval of the version to be published. Authors should meet conditions 1, 2 and 3.

Any person who cannot be shown to have made a substantial contribution to the article cannot be listed as an author in the final version. The name of any person who is deemed to have made a minor contribution can, however, appear in the Acknowledgments section of the article.

Please complete the table below to indicate the contributions of all named authors to the manuscript.

Author Full Name:	Specification of Contribution to the Manuscript:
Huan Wang	Writing, Review and Revise
Ling Zhang	Writing, Review and Revise
Qiuhua Luo	Review and Revise
Guiling Wang	Study concept, Design, Writing, Review and Revise

Please complete the table below to indicate the contributions of all named authors to the figures.

Figure 1:
Huang Wang drawed the figure according to the referrences.Guiling Wang revised the figure.

Figure 2:
Ling Zhang searched data from databases and assmebled the figure. Guiling Wang revised the figure.

Figure 3:
Ling Zhang searched data from database and assmebled the figure. Guiling Wang revised the figure.

Figure 4:
Qiuhua Luo drawed the figure according to the referrences.

Figure 5:
Huan Wang searched data from database and assmebled the figure.

Figure 6:

Signed for and on behalf of the Author(s):	Print Name:	Date:
Guiling Wang	Guiling Wang	6/25/2021
